# PET and Active Coating Based on a LDH Nanofiller Hosting p-Hydroxybenzoate and Food-Grade Zeolites: Evaluation of Antimicrobial Activity of Packaging and Shelf Life of Red Meat

**DOI:** 10.3390/nano9121727

**Published:** 2019-12-03

**Authors:** Valeria Bugatti, Luigi Vertuccio, Federica Zuppardi, Vittoria Vittoria, Giuliana Gorrasi

**Affiliations:** 1 Department of Industrial Engineering, University of Salerno, via Giovanni Paolo II, 132, 84084 Fisciano (SA), Italy; vbugatti@unisa.it (V.B.); lvertuccio@unisa.it (L.V.); vvittoria@unisa.it (V.V.); 2 Nice Filler s.r.l., via Loggia dei Pisani, 25, 80133 Napoli, Italy; federica.zuppardi@nicefiller.it

**Keywords:** layered double hydroxides, zeolites, active coating, antimicrobial, food packaging

## Abstract

Layered double hydroxide (LDH) nanofillers were considered as hosts of p-hydroxybenzoate as an antimicrobial molecule for active coating. A food grade resin with LDH-p-hydroxybenzoate and two different types of food grade zeolites was used to prepare active coatings for Polyethylene terephthalate (PET) trays. The release kinetics of the active molecule were followed using UV spectrophotometry and the experimental results were analyzed with the Gallagher–Corrigan model. The thermal properties of the coating mixtures and the PET coating were analyzed and found to be dependent on the coating’s composition. On the basis of CO_2_ transmission rate and off-odors tests, the best coating composition was selected. Global migration in ethanol (10% v/v), acetic acid (3% w/v), and vegetable oil, and specific migration of p-hydroxybenzoic acid revealed the suitability of the material for food contact. Antimicrobial tests on the packaging demonstrated a good inhibition against *Salmonella* spp. and *Campylobacter* jejuni. Red meat was packed into the selected active materials and results were compared to uncoated PET packaging. Color tests (browning of the meat) and analysis of *Enterobacteriaceae* spp. and total viable count evolution up to 10 days of storage demonstrated the capability of the considered active packaging in prolonging the shelf life of red meat.

## 1. Introduction

Active packaging is a research area with important effects on the circular economy and consumer welfare. A packaging is “active” if the material, along with the basic protection, performs additional functions with respect to the containment. The applications in the food field are related to the goal of improving the shelf life, safety, or quality of packaged products. Active packaging, with antimicrobial activity for food products, can be divided into several categories: (i) volatile antimicrobial substances added into packaging, which are often enclosed separately in sachets or pads attached to the internal part of the package; (ii) antimicrobial agents incorporated into the bulk polymer (either using extrusion of casting); (iii) antimicrobials immobilized by ionic or covalent linkages to polymers; (iv) coating of polymer surfaces with antimicrobials; (v) use of polymers that are inherently antimicrobial (i.e., chitosan, poly-L-lysine, polyurethane with quaternary ammonium) [1,2,3]. In recent years, coating strategies for incorporating antimicrobials into packaging have been gaining great interest. If the antimicrobial product is heat-sensitive, methodologies used to realize an active packaging that involve high temperatures can compromise its activity. Using there techniques, the antimicrobial agent can be coated directly onto the film material before applying it to foods. Another advantage over bulk incorporation is the fact that the incorporation of the active agent on the product contact side can minimize the amount used, and consequently the cost of the active molecules required to impart efficacy to the system can be contained [4,5]. In recent years, layered double hydroxides (LDH), or hydrotalcite-like compounds, have received considerable attention as hosts of active molecules as delivery vehicles, due to their anion exchange capability. The general formula of such anionic clays is [M(II)_(1−x)_M(III)_x_(OH)_2_](A_x/n_)^−^·mH_2_O, where M(II) is a divalent cation, such as in Zn^2+^, Mg^2+^, Ni^2+^, Co^2+^, or Cu^2+^; M(III) is a trivalent cation, such as in Al^3+^, Fe^3+^, Ga^3+^, or Cr^3+^; and A^n-^ is an anion of charge, such as in CO_3_^2−^, NO_3_^−^, Cl^−^, or an organic anion [6,7]. The ionically bonded organic active molecules either improve the compatibility with the polymer matrix or slow their release activity in specific environments for targeted applications. The release occurs via a de-intercalation process, which consists of anion exchange displacement reactions [8,9]. Poly(ethylene terephthalate) (PET) is one of the most used engineering polymers worldwide; in fact, around 50 million tons of PET are produced each year because of its applications, from packaging to fiber production. In spite of this large use of PET in manufacturing, its consumptions in the last twenty years significantly increased over other alternative packaging materials (glass, aluminium, paper). In addition, there is growing demand for a new generation of bio-plastics for packaging because of fluctuating oil prices and greater consumer awareness of sustainability issues. Non-petroleum-derived PET is becoming one of the most important new bioplastics being targeted. The incorporation of antimicrobial species into PET using melt extrusion is limited because of the high melting point of such materials, which can cause thermal degradation of the organic molecules during the processing. An interesting alternative could be the utilization of an active coating in PET packaging using materials filled with antimicrobial molecules, in order to avoid thermal stress to the organic molecules and reduce the number of effective molecules. In this paper, we report the preparation of active packaging (trays) based on PET coated with a food-grade resin, filled with a LDH hosting para-hydroxy benzoate as an antimicrobial, and two different types of food-grade zeolites as odor and volatile organic compound scavengers, along with the CO_2_ transmission rate and inhibition rate of bacteria spoilage. The fresh meat’s shelf life was evaluated in terms of color change and inhibition against *Enterobacteriaceae* spp. and total viable count. 

## 2. Experimental

### 2.1. Materials

Poly(ethylene terephthalate) (PET) (Echantillon PCV0000 MOD. 85030AF) used for the top of the trays was supplied by Coopbox, Reggio Emilia (Italy), in film form 30 μm thick. Poly(ethylene terephthalate) (PET) (B.PET TRA S2 F740 S350, 2157400350) used for the bottom of the trays was supplied by Aristea spa, Salerno (Italy), in laminae form 350 μm thick. These laminae were then processed in trays. The active filler, based on LDH intercalated with antimicrobial para-hydroxy benzoate anion (listed in EC-Directive 10/2011), was produced by Nicefiller Ltd., a start-up company owned by the University of Salerno (Italy), under the trade name N6O6^®^. The synthesis was conducted according to a previously reported procedure [10]. Zeolites (Zeoflair 100 e Zeoflair 810) with the trade name ZEOflair^®^ were manufactured by Zeochem d.o.o., Karakaj 105a, 75400 Zvornik, Bosnia and Herzegovina. Zeochem d.o.o. is ISO 9001:2015 certified for food contact. The resin used for coating was a water-based paint normally used for food packaging (Inx 1-7801-6000, solid content 43 ± 2%, viscosity 20 s at 20 °C) purchased from Inx srl (Lodi, Italy). Its constituents are in accordance with the EC-Directive 2002/72, including amendments. The active filler (N6O6) at 60 wt% was mixed either with Zeoflair 100 at 40 wt% and coated on PET (sample named PET/Active Zeo 100) or with Zeoflair 810 at 40 wt% and coated on PET (sample named PET/Active Zeo 810) using high-energy ball milling at ambient temperature for 30 min at 450 rpm. An active sample of PET coated with N6O6 without zeolites was also produced (named PET Active). The composite filler weight was 5 ± 0.5 g/m^2^ on dry resin. 

### 2.2. Methods

X-ray diffraction (XRD) patterns were taken in reflection with an automatic Bruker diffractometer D8 (Karlsruhe, Germany), using nickel-filtered Cu Kα radiation (Kα = 1.54050 Å) and operating at 40 kV and 40 mA, with a step scan of 0.05° of 2 ϑ and 3 s of counting time.

Thermogravimetric analyses (TGA) were carried out from 30 to 800 °C (heating rate of 10 °C/min) under air flow, using a Mettler TC-10 thermo-balance (Mettler-Toledo GmbH, Greifensee, Switzerland). 

The release kinetics of para-hydroxy benzoate were followed using a Shimadzu UV-2401 PC spectrometer (Shimadzu, Kyoto, Japan). The tests were performed using 4 cm^2^ rectangular specimens placed into 25 mL physiological solution and stirred at 100 rpm in an orbital shaker (VDRL MOD. 711+, Asal S.r.l., Milan, Italy). The release medium was withdrawn at fixed time intervals and replenished with fresh medium. The considered band was 255 nm.

Permeability to carbon dioxide was determined according to American Society for Testing and Materials (ASTM) standard 2476-13 by using a Mocon Permatran C 4/41 automatic equipment (Minneapolis, MN 55428 USA) (n. Series 0806AY066). The operating conditions included a temperature and humidity of 23 °C and 0% relative humidity (RH); the film area was 50 cm^2^. Samples were conditioned for 48 h in a desiccator containing calcium chloride and subsequently in the instrument for 6 h; the orientation of the samples was with the untreated side facing towards humidity. The partial CO_2_ pressure was 1 atm and the carrier flow was 10 cc/min. The calibration was carried out with Mocon standard.

Sensory tests were conducted according to the Renault D49 3001-E directive (D49 3001/-C. 1996). The Renault test method involved evaluation of emitted odors, intensity of internal equipment parts, and global odor characterization. 

In vitro testing of bacteria inhibition was done on the PET/active Zeo 810 against *Salmonella* spp. and *Campylobacter* jejuni, following ISO 22196:2011.

Antimicrobial tests were carried out on meat packed in untreated PET packaging and PET/active Zeo 810. The analysis time was 10 days of storage at a temperature of 7 °C. Vacuum/gas packaging was 70% O_2_ and 30% CO_2_ (nominal values). The evolution of gas in the head space showed no differences between the standard thesis and antimicrobial thesis. The analysis of the evolution of total viable count and *Enterobacteriaceae* spp. was conducted. For microorganisms isolation, 10 g of red meat from each sample was aseptically collected after 3, 6, and 10 days of storage and added to 90 mL of saline peptone water. The mixture was homogenized for 1 min in a stomacher 400 (Lab Blender, Seward Medical, London, UK), and 1mL of homogenate was subjected to serial dilutions in the same diluent. Aliquots of 0.1 mL of different dilutions were spread onto the culture media, which were selective for each type of organism (all from Oxoid). Microorganisms were enumerated with the method based on the count of colony forming units (CFU), by using 25–250 CFU plates as the range of countable colonies to limit the error due to variability (1, 2, 3). Total viable count was evaluated by unselective plate count agar (PCA) incubating at 30 °C for 48 h (UNI EN ISO 4833-1:2013/UNI EN ISO 4833-2:2013/Cor.1:2014). *Enterobacteriaceae* spp. was evaluated by RAPID *Enterobacteriaceae* spp. medium, high-performance medium for the enumeration of *Enterobacteriaceae* spp. in food, and with environmental samples at 30 °C for 24 h according to the ISO 16140 standard and validated by Association of Official Agricultural Chemists (AOAC). The reported microbiological values are the average of three replicates.

Color tests on fresh meat, either packed in untreated PET packaging or PET/active Zeo 810, were performed in the CIE-Lab space. The color data were processed by calculating the value of “ΔE” or global color change for each sample. A Minolta CM-2002 spectrophotometer (Cinisello Balsamo (MI)) with an integrating sphere, d/8 (diffuse illumination/8° viewing angle) geometry, and 8 mm diameter circular aperture was used to record the meat spectra from 400 to 700 nm at 10 nm intervals, and to obtain the CIE-Lab color coordinates L*, a*, b*, chroma C*, and hue angle hab for the D65 illuminant and a 10° standard observer. In order to avoid pillowing effects and to prevent dirtying the instrument’s internal integrating sphere, all measurements were performed through a protecting glass cover supplied with the spectrophotometer (cover set CM-A40). Measurements were performed directly on the meat surface without the film. Calibration was performed with the glass, using a white calibration plate (CM-A21) and a light trap (Zero Calibration Box CM-A32). Each measurement was the average of five different readings in five non-overlapping zones of each slice, changing the instrument orientation each time to reduce the effect of possible inhomogeneities such as fat and blood. Measurements were taken after 3, 6, and 10 days, thus allowing the evolution over time of myoglobin forms and providing a representative set of pigment concentrations. Color parameters (L*, a*, and b*) were measured on 3 random points on the peel surface of slices for each replicate with a colorimeter (CR-400, Konica Minolta, Osaka, Japan) in the reflectance mode and in the CIE L* a* b* color scale. The colorimeter was calibrated with a standard reference, with values for L*, a*, and b* corresponding to 97.55, 1.32, and 1.41, respectively. Color was expressed as total color variation (ΔE = $\sqrt{{\left(L_{0}^{*}-L_{t}^{*}\right)}^{2}+{\left(a_{0}^{*}-a_{t}^{*}\right)}^{2}+{\left(b_{0}^{*}-b_{t}^{*}\right)}^{2}}$) calculated from primary L*, a*, and b* readings. 

Overall migration tests were performed on the PET/active Zeo810 sample according to the following procedure: lamina specimens with 1 dm^2^ of surface area (10 cm × 10 cm, 0.10 mm thickness) were put into contact with 100 mL simulant (preconditioned at 40 °C) in a borosilicate glass tube sealed with a screw cap internally layered with Teflon^®^. The obtained surface/volume ratio was 10 dm^2^/L. Migration tests after contact for 10 days at 40 °C were performed using A (ethanol at 10%), B (acetic acid at 3%), and D2 (vegetable oil) as simulants. The overall migration test was performed on different aliquots from the same contact sample. The overall migration results were calculated by using 6 dm^2^/kg food (6 dm^2^/L simulant) at a conventional EU surface/volume ratio. A known aliquot of the simulant from the contact solution was transferred into a weighted (s = 0.1 mg) quartz capsule and evaporated to dryness until it reached a constant weight. From the differences between the weights, the overall migration was derived in accordance to EN 1186 Migration Testing for Food Contact Materials. The data were averaged from five samples.

## 3. Results and Discussion

**Figure 1:**
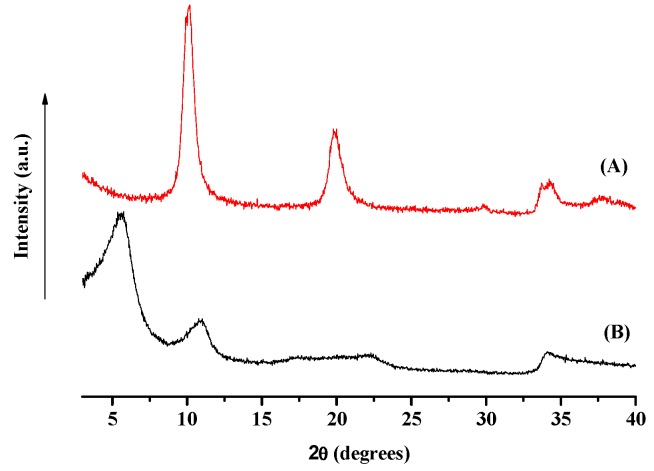
X-ray diffraction (XRD) analysis of pristine layered double hydroxide (LDH) in nitrate form (**A**) and LDH hosting para-hydroxybenzoate anion (**B**).

Figure 1 shows the pristine LDH in nitrate form (A), with the basal spacing at 10.2° of 2 ϑ (d = 8.6 Å). The successful intercalation of the para-hydroxybenzoate anion into LDH layers is demonstrated by the fact that the basal spacing is increased; in fact, the interlayer distance is revealed by the peak at 5.6° of 2 ϑ (d = 14.86 Å).

**Figure 2:**
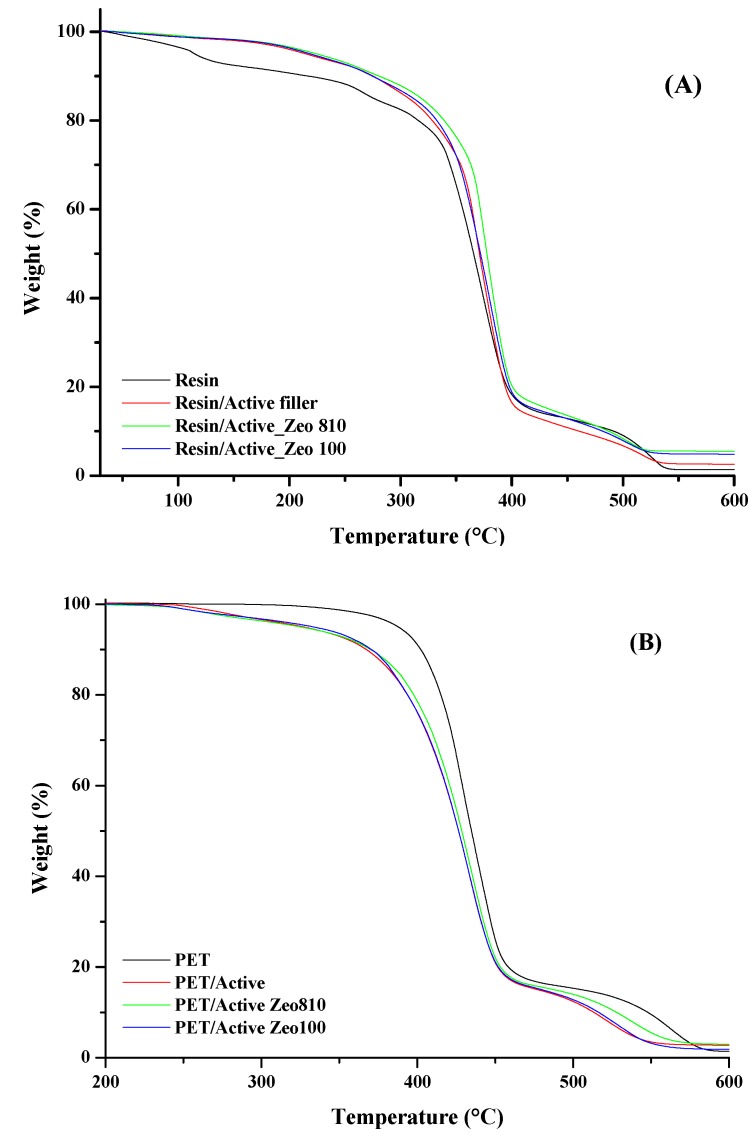
Thermogravimetric analyses (TGA) evaluated on coating materials (**A**) and on poly(ethylene terephthalate) (PET) and PET-coated trays (**B**).

Figure 2 shows the TGA analyses evaluated on coating materials (Figure 2A) and on PET and PET-coated trays (Figure 2B). The starting acrylic resin shows four degradation steps. The first one, at about 130 °C, is related to the loss of internal water. The second one, at around 290 °C, corresponds to residual acrylic monomers and some oligomers. The third one (the main weight loss), at about 365 °C, corresponds to the decomposition of the backbone chains. The fourth one, at 490 °C, is due to the volatilization of low-molecular-weight organic compounds. The introduction of active filler and zeolites into the pristine resin produces a better thermal resistance, in particular for the sample filled with Zeo 810. PET shows a two-step weight loss stage during the heating process, including a major loss (centered at about 434 °C) due to the random chain scission and a minor loss (centered at about 550 °C) due to the production of gaseous products (i.e., acetaldehyde, carbon dioxide, carbon monoxide, water, and other volatile compounds with acid and anhydride groups). The presence of the coating on the PET surface allows anticipation of the thermal degradation of the polymer across the whole investigated temperature range. PET/Active Zeo 810 results show better resistance, in particularly in the second degradation step.

**Figure 3:**
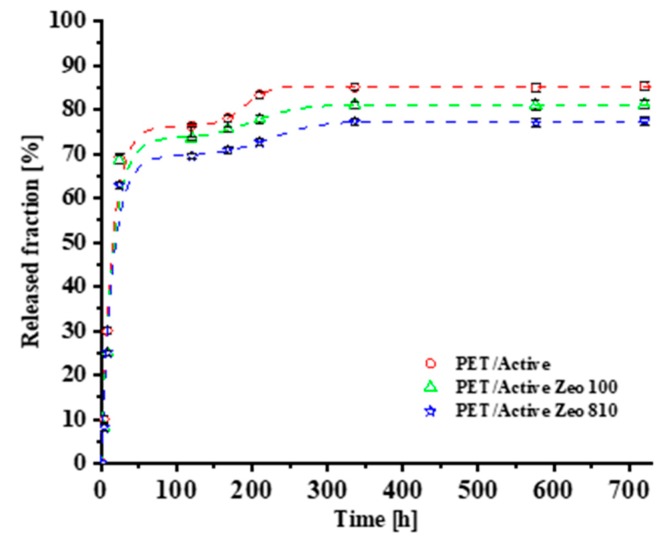
Released fraction (%) of para-hydroxybenzoate as a function of time for PET/Active, PET/Active Zeo 100, and PET/Active Zeo 810 samples.

Figure 3 reports the released fraction of para-hydroxybenzoate as a function of time for PET/Active, PET/Active Zeo 100, and PET/Active Zeo 810 samples.

The experimental data were fitted with the Gallagher–Corrigan model [11]. The model describes the drug release phenomenon by two-stage kinetics: the first one is characterized by the first order kinetics, which reflects the diffusion controlled dissolution of the drug from the film; the second one describes the dependence of the drug delivery on the chain relaxation [12,13,14,15]. The model provide the total fraction of drug released as a function of time, according to the following equation:

(1) $$f_{t}=\;f_{b}*\left(1-e^{-k_{1}t}\right)+\left(f_{tmax}-f_{b}\right)\left(\frac{e^{k_{2}\left(t-t_{2max}\right)}}{1+e^{k_{2}\left(t-t_{2max}\right)}}\right),$$

where *f_t_* is the accumulative drug release percentage at time *t*, *k*_1_ is the first order release constant, *k*_2_ is the second stage release constant, *f_b_* is the accumulative drug release percentage during the first step, *f_tmax_* is the maximum drug release percentage during the whole process, and *t*_2*max*_ is the time at which the drug release rate reaches the maximum. The correlation coefficient (R^2^) was used as an indicator of the best fit for the considered model. The experimental data and the related fitting model are shown in Figure 1, while the kinetic parameters obtained from Equation (1) are shown in Table 1. The introduction of Zeo 100 and Zeo 810 causes a reduction of both *f_b_* and the maximum value of released para-hydroxybenzoate, with a consequent increase of *t*_2*max*_. The presence of both zeolites appears to slow the drug release, as demonstrated by the values of *K*_1_ and *K*_2_. Such phenomena are probably due either to the capability of zeolites to entrap the active molecule, or to the further tortuosity of the pathway that lowers the molecules’ diffusion. The above described phenomena are more evident in the case of PET/active Zeo 810.

The carbon dioxide transmission rate was evaluated on the films that covered the trays. It is known that CO_2_ is used as a protective gas in food packaging. It has an oxidation-inhibiting and growth-inhibiting effect on most aerobic bacteria and molds. The gas is frequently used to increase the shelf life of foods. The shelf life of packaged or stored food is normally longer with a higher CO_2_ content. Table 2 reports the experimental results. It is evident that the addition of the zeolites lowers the CO_2_ transmission rate, in particular for the PET/Active Zeo 810 sample.

**Figure 4:**
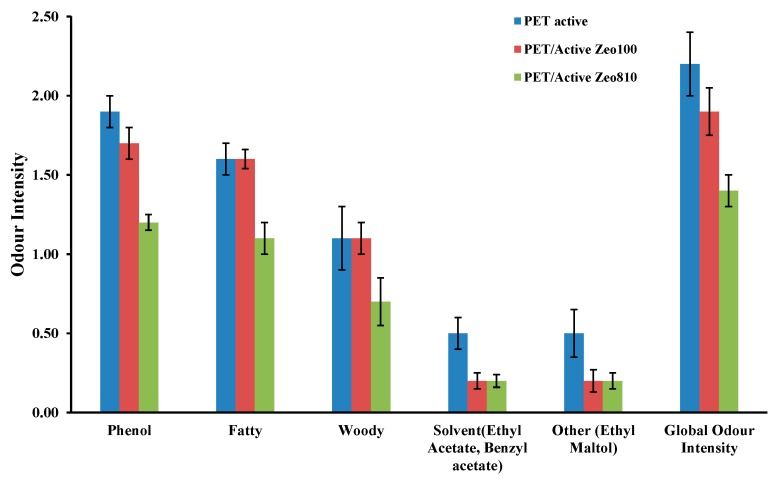
Odor tests performed on all the films used as tray covers.

Odor tests were performed on all the films used as covers for the trays. The sensory test method organizes the olfactive space according to 6 axes (amine, hesperidic, terpenic, sulphured, pyrogenous, and sweet). Descriptors (reference molecules diluted in alcohol) are used to characterize the odors and can be seen as the alphabet used to analyze and describe odors. The description of odors obtained with the reference system was both qualitative (presence or absence of the descriptors) and quantitative. For each descriptor, an overall olfactory intensity and an intensity value were given (relative scale). Sensory profiles obtained by such characterization methods may be seen as characteristic signatures of the described product. The odor test was performed after heating the samples (0.6 g in 60 mL vials) at 70 °C for 2 h. Figure 4 shows the experimental results. The addition of zeolites to the active filler had a positive impact on the reduction of the global intensity level of the samples. This effect seems to be prominent in the PET/active Zeo 810 sample. In fact, this sample shows the lowest intensity levels for the phenol, fatty, woody, and global odor intensity parameters.

Taking into account the described results, we selected the PET/active Zeo 810 sample as an active packaging prototype for fresh meat. We also performed migration tests and in vitro antimicrobial analysis on the base of the trays (the part in contact with the meat) in order to demonstrate that the considered materials are suitable for food contact. Then, on the packed fresh meat, we carried out microbiological assays and color evaluation as a function of time. Table 3 shows the global migration and specific migration of p-hydroxybenzoic acid, evaluated on the PET/active Zeo 810 material, in three different food simulants (ethanol (10% v/v), acetic acid (3% w/v), and vegetable oil) following the directives UNI EN 1186-1: 2003 and UNI EN 1186-9: 2003. The experimental results, in compliance with the migration limits, demonstrate the suitability of the considered material for food contact.

Inhibition tests against two strains of Gram-negative bacteria were evaluated on the PET/Active Zeo 810 trays. The test microorganism was prepared by growth in a liquid culture medium. Two representative microorganisms were considered in the present case: *Salmonella* spp. and *Campylobacter* jejuni. The suspension of the test microorganism was standardized by dilution in a nutritive broth (this afforded microorganisms the potential to grow during the test). Control and test surfaces were inoculated with microorganisms in triplicate and then the microbial inoculum was covered with a thin, sterile film. All microbiological assays run were performed with the necessary parallel controls to provide adequate comparisons at both the start of the test as well as after the contact time, which in this case was 24 h. Microbial concentrations were determined at “time zero” by elution followed by dilution and plating. A control was run to verify that the neutralization–elution method effectively neutralized the antimicrobial agent in the antimicrobial surface being tested. Inoculated and covered control and antimicrobial test surfaces were allowed to incubate undisturbed in a humid environment for 24 h. After incubation, microbial concentrations were determined. The reduction of microorganisms was calculated relative to initial concentrations and the control surface. Table 4 reports the experimental conditions and results. A significant antibacterial activity from the considered material is evident with both strains.

**Figure 5:**
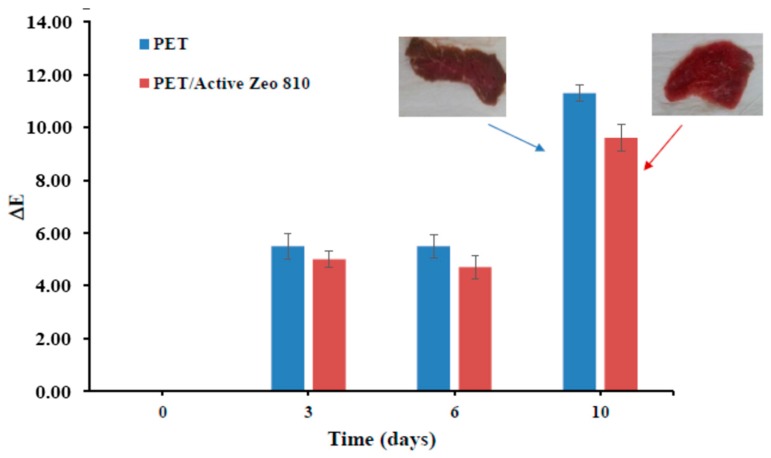
Color evolution (browning) on red meat packed in untreated PET and in PET/Active Zeo 810.

PET/Active Zeo 810 was used as packaging for red meat, and an untreated PET tray was used as reference packaging. Analysis of color and antimicrobial tests were carried out on meat pieces as a function of time up to ten days of storage. The analyzed red meat was beef loin in slices. Figure 5 shows the evolution of color in the CIE-Lab space. The color data were processed by calculating the value of “ΔE” or “global color change” for each sample. The results demonstrate that the color variation (browning, see photos at 10 days) is more rapid in the standard untreated packaging compared to the antimicrobial treated material.

**Figure 6:**
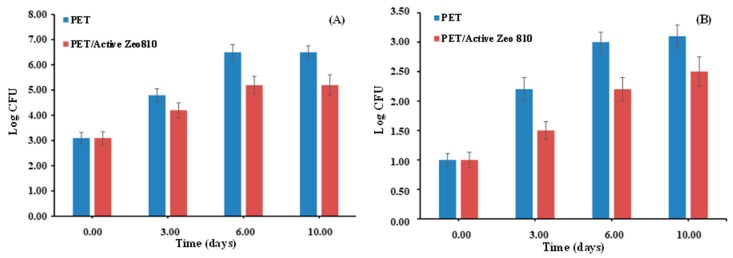
Evolution total viable count (**A**) and *Enterobacteriaceae* spp. (**B**) on red meat packed in untreated PET and in PET/Active Zeo 810.

Figure 6 shows the evolution of total viable count (Figure 6A) and *Enterobacteriaceae* spp. (Figure 6B). In both cases an improved antibacterial activity is evident from the active packaging with respect to the packed meat. The bacteria inhibition at any stored time is one log lower in the active packaging compared to the untreated PET. These results confirm that the investigated active coating may exert an inhibitory effect on the microorganisms responsible for spoilage phenomena, extending the shelf life of the packed red meat [16].

## 4. Concluding Remarks

An active coating was prepared using a food-grade acrylic resin filled with a LDH nanofiller hosting antimicrobial para-hydroxybenzoate (listed in EC-Directive 10/2011). Two types of commercial food-grade zeolites (Zeo 100 and Zeo 810) were added to the active coating mixture. The introduction of active filler and zeolites into the pristine resin produced a better thermal resistance, in particular for the sample filled with Zeo 810. The presence of the coating on the PET surface allowed anticipation of the thermal degradation of the polymer across the whole investigated temperature range. PET/Active Zeo 810 resulted in better resistance, in particular at higher degradation temperatures. On the basis of off-odor tests and CO_2_ transmission rate, the packaging coated with active Zeo 810 was selected. Analyses of overall migration were conducted on this material in ethanol (10% v/v), acetic acid (3% w/v), and vegetable oil, along with specific migration of p-hydroxybenzoic acid, revealing its suitability for food contact. *Salmonella* spp. and *Campylobacter jejuni* were tested and PET/Active Zeo 810 revealed a significant inhibition against these two Gram-negative bacteria strains. Shelf life was evaluated on red meat packed either in PET/Active Zeo 810 or in uncoated PET. Color tests (browning of the meat) and analysis of *Enterobacteriaceae* spp. and total viable count up to 10 days of storage demonstrated the capability of the considered active packaging in prolonging the shelf life of red meat.

## Figures and Tables

**Table 1:** Kinetic parameters derived from Equation (1).

Sample	*f_b_* (%)	*K*_1_ (h^−1^)	*K*_2_ (h^−1^)	*t*_2*max*_ (h)	R^2^
PET/active	76	6.92 × 10^−2^	6.58 × 10^−2^	190	0.988
PET/Active Zeo 100	74	6.63 × 10^−2^	3.37 × 10^−2^	202	0.979
PET/Active Zeo 810	70	6.59 × 10^−2^	3.09 × 10^−2^	223	0.985

**Table 2:** Carbon dioxide transmission rate (TR) at 23 °C.

Sample	CO_2_ TR [g/(m^2^ × 24 h)]
PET/Active	824 ± 15
PET/Active Zeo 100	816 ± 20
PET/Active Zeo 810	778 ± 22

**Table 3:** Global migration for PET/Active Zeo 810 in ethanol (10% v/v), acetic acid (3% w/v), and vegetable oil (UNI EN 1186-1: 2003 and UNI EN 1186-9: 2003), and specific migration of p-hydroxybenzoic acid.

Simulant Used	A—Ethanol at 10%	B—Acetic Acid at	D2—Vegetable Oil	Limits
	(v/v)	3% (w/v)		
Temperature of the test	40 °C	40 °C	40 °C	
Contact time	10 days	10 days	10 days	
Global migration	<1.0 mg/dm^2^	1.4 mg/dm^2^	7.7 mg/dm^2^	10 mg/dm^2^
p-hydroxybenzoic acid (cas 99-96-7): specific migration	1.8 mg/kg	2.1 mg/kg	0.8 mg/kg	60 mg/kg

**Table 4:** Inhibition of Gram-negative strains on PET/Active Zeo 810.

Gram-Negative Strain	*Campylobacter jejuni*—ATCC 33291	*Salmonella* spp. *enterica*
		*typhimurium*—ATCC
		14028
Sample size	50 × 50 (mm × mm)	50 × 50 (mm × mm)
Sample thickness	1.0 mm	1.0 mm
Inoculum volume	0.4 mL	0.4 mL
U_o_: number of bacteria available in the inoculum	300 000	120 000
U_t_: bacteria count recovered from non-treated samples 24 h after inoculation	3.8 (Log)	4.3 (Log)
A_t_: bacteria count recovered from samples treated 24 h after inoculation	≤0.4 (Log)	1.6 (Log)
Antibacterial activity (R)	>3.8	2.6
R = (U_t_ − U_o_) − (A_t_ − U_o_)		
ISO 22196:2011		

## References

[1] Sung S.Y., Sin L.T., Tee T.T., Bee S.T., Rahmat A., Rahman W., Tan A.C., Vikhraman M. (2013). Antimicrobial agents for food packaging applications. Trends Food Sci. Technol..

[2] Han J.W., Ruiz-Garcia L., Qian J.P., Yang X.T. (2018). Food packaging: A comprehensive review and future trends. Compr. Rev. Food Sci. Food Saf..

[3] Gan I., Chow W.S. (2018). Antimicrobial poly (lactic acid)/cellulose bionanocomposite for food packaging application: A review. Food Packag. Shelf Life.

[4] Bastarrachea L.J., Wong D.E., Roman M.J., Lin Z., Goddard J.M. (2015). Active Packaging Coatings. Coatings.

[5] Gorrasi G., Bugatti V., Tammaro L., Vertuccio L., Vigliotta G., Vittoria V. (2016). Active coating for storage of Mozzarella cheese packaged under thermal abuse. Food Control.

[6] Costantino U., Marmottini F., Nocchetti M., Vivani R. (1998). New Synthetic Routes to Hydrotalcite-Like Compounds Characterisation and Properties of the Obtained Materials. Eur. J. Inorg. Chem..

[7] Costantino U., Nocchetti M., Gorrasi G., Tammaro L., Lagarón J.-M. (2011). 3-Hydrotalcites in nanobiocomposites. Multifunctional and Nanoreinforced Polymers for Food Packaging.

[8] Bugatti V., Gorrasi G., Montanari F., Nocchetti M., Tammaro L., Vittoria V. (2011). Modified layered double hydroxides in polycaprolactone as a tunable delivery system: In vitro release of antimicrobial benzoate derivatives. Appl. Clay Sci..

[9] Gorrasi G., Bugatti V. (2016). Edible bio-nano-hybrid coatings for food protection based on pectins and LDH-salicylate: Preparation and analysis of physical properties. LWT Food Sci. Technol..

[10] Gorrasi G., Bugatti V. (2016). Mechanical dispersion of layered double hydroxides hosting active molecules in polyethylene: Analysis of structure and physical properties. App. Clay Sci..

[11] Gallagher K.M., Corrigan O.I. (2000). Mechanistic aspects of the release of levamisole hydrochloride from biodegradable polymers. J. Control. Release.

[12] Dunne M.M., Ramtoola Z., Corrigan O.I.J. (2009). Fluphenazine release from biodegradable microparticles: Characterization and modelling of release. Microencapsul.

[13] He J., Zhong C., Mi J. (2005). Modeling of drug release from bioerodible polymer matrices. J. Drug Deliv..

[14] Milallos R.G., Alexander K., Riga A. (2008). Investigation of the interaction between acidic, basic, neutral, and zwitterionic drugs with poly-L-lactic acid by thermal and analytical methods. J. Therm. Anal. Calorim..

[15] Bugatti V., Vertuccio L., Zara S., Fancello F., Scanu B., Gorrasi G. (2019). Green pesticides based on cinnamate anion incorporated in layered double hydroxides and dispersed in pectin matrix. Carbohydr. Polym..

[16] Sun X.D., Holley R.A. (2012). Antimicrobial and Antioxidative Strategies to Reduce Pathogens and Extend the Shelf Life of Fresh Red Meats. Compr. Rev. Food Sci. Food Saf..

